# Double cortex syndrome (subcortical band heterotopia): A case report

**DOI:** 10.1016/j.radcr.2022.11.021

**Published:** 2022-12-05

**Authors:** Faiza Afzal, Shehroze Tabassum, Aroma Naeem, Farhan Naeem, Rana Uzair Ahmad

**Affiliations:** King Edward Medical University, Nila Gumbad Chawk, Lahore, Punjab, 54000, Pakistan

**Keywords:** Developmental delay, Band heterotopia of brain, Seizures

## Abstract

Double cortex syndrome is an uncommon familial syndrome with X-linked dominant inheritance and most commonly presents with developmental delay and seizures. We present a case of a 14-year-old girl who came to neurology department of the hospital with severe generalized tonic-clonic fits and loss of consciousness. The mother of child gave history of uneventful antenatal period and labor. There was history of immediate cry and normal APGAR score. She was achieving milestones normally until at the age of 3 years when she suffered decline in her speech and vision. She had problems with learning with lack of concentration during her schooling. Physical examination was also unremarkable. Her lab values including complete blood count, serum calcium, and arterial blood gas tests, all were within normal limits. Electroencephalogram showed significant changes suggestive of epilepsy. Magnetic resonance imaging of brain showed continuous band of gray matter that was located deep and paralleling the cortex in both cerebral hemispheres suggestive of band heterotopia or double cortex syndrome. She was discharged and prescribed antiepileptics; and was advised regular outpatient follow-up.

## Introduction

Neuronal migration abnormality is a broad term which encompasses terms such as lissencephaly, pacchygyria, heterotopias, schizencephaly, hemimegalencephaly, and fibrous cortical dysplasia. Cortical development of fetus includes 3 main stages including proliferation, migration, and organization which are inseparable. It has been theorized that delay at any level of these stages constitutes the etiology of several congenital anomalies. Insult to the fetal brain, whether genetic, toxic, infectious, or ischemic, can result in growth arrest of the developing cortex. Band heterotopia, also known as double cortex syndrome, is a rare form of diffuse gray matter heterotopia that affects mostly women due to the fact that genetic abnormality is of *DCX* gene [1], located on the long arm of chromosome X [2]. Seizures and developmental delay are the most common presentations, usually manifesting within the initial years of life [3]. Seizures may even advance to refractory seizures [4]. Neurological and physical examination can be normal in some cases; however, hypotonia, dysarthria, poor fine motor control, or, in rare cases, a pyramidal syndrome may even be develop in patients [5]. The main diagnostic modality is magnetic resonance imaging (MRI), which reveals the characteristic continuous subcortical heterotopic band isointense to gray matter in all sequences [6]. The band can be of different thickness and size. The overlying cortical mantle may appear normal on MRI or may demonstrate abnormalities ranging from agyria to pachygyria [7]. The severity of cortical anomaly can be related to the heterotopic band thickness. The thickness of the band of heterotopic gray matter relates to the shallowness of the sulci in the overlying cortex [7]. We present a case of a 14-year-old girl diagnosed with a rare syndrome known as double cortex syndrome.

## Case report

A 14-year-old girl presented with the complaints of generalized tonic-clonic fits, upward gaze, tongue biting, and loss of consciousness for few seconds in neurology department of the hospital. Her birth was at term by normal delivery and is the first child of her parents. Patient had an immediate cry with good APGAR score at birth. Throughout her infancy and even as a toddler, she achieved her milestones normally and was an active child. Suddenly, she stopped talking at the age of 3 years due to difficulty in articulation along with history of recurrent falls during walking or even standing. There was a further decline observed in her condition and she developed seizures and reduced vision at the age of 6 years. During schooling, she had learning problems due to lack of concentration in her studies. Her computed tomography (CT) scan was done when she was around 7 years of age which was unremarkable. All her laboratory reports including complete blood count and serum calcium level were normal. She was not taking any medication for fits until when she developed generalized tonic-clonic seizure. She was hypotonic otherwise her physical and neurological examination was unremarkable. Her electroencephalogram done recently showed abnormal spike waves. MRI was done which showed the characteristic subcortical heterotopic band with signal intensity similar to gray matter on all sequences. The overlying cortical mantle and corpus callosum were unremarkable. She was started on antiepileptics and discharged later with advice of proper follow-ups on outpatient department basis.

**Fig. 1:**
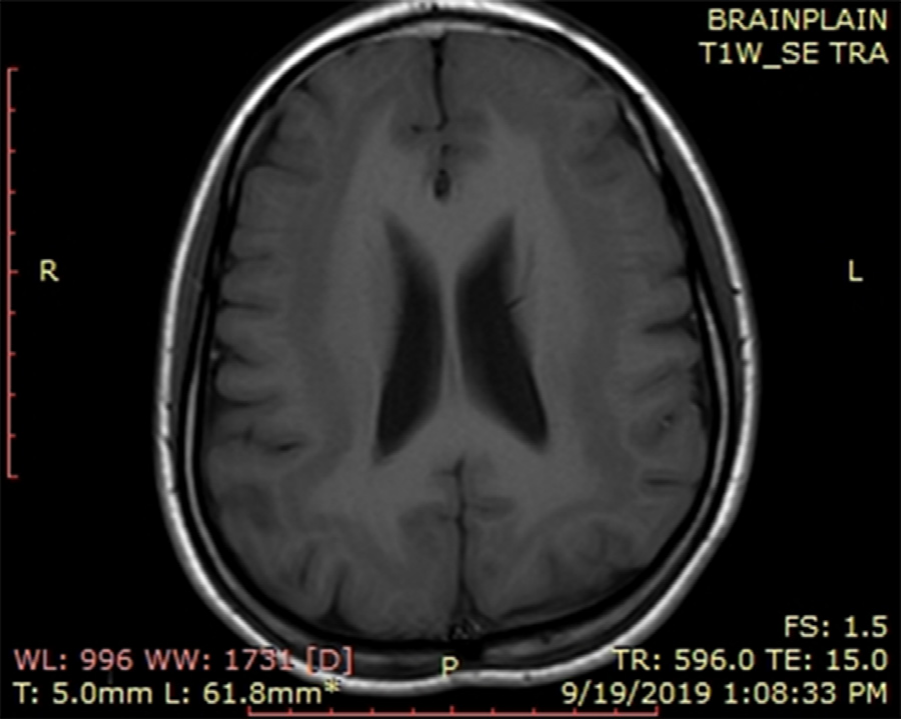
MRI brain plain T1WI showing continuous subcortical band of heterotopic grey matter.

**Fig. 2:**
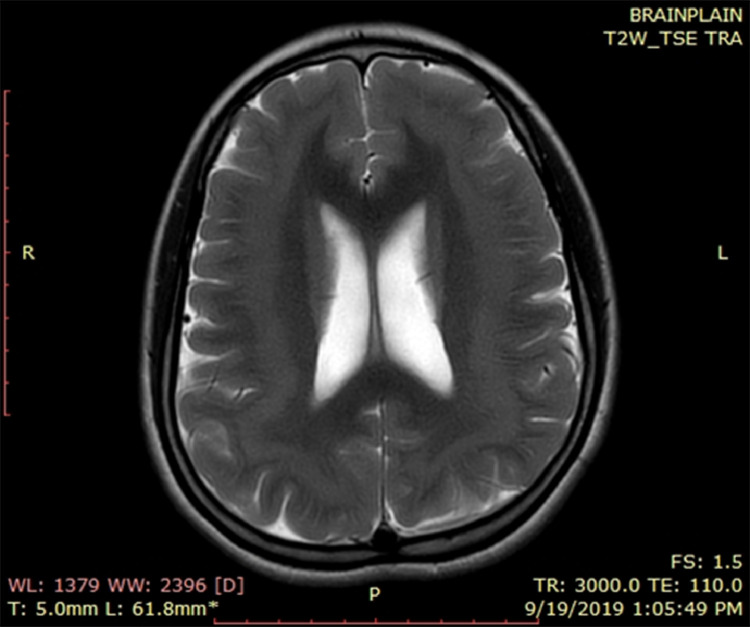
MRI brain plain T2WI showing continuous subcortical band of heterotopic grey matter.

MRI brain plain T1WI (Fig. 1) and T2WI (Fig. 2) show continuous subcortical band of heterotopic gray matter.

**Fig. 3:**
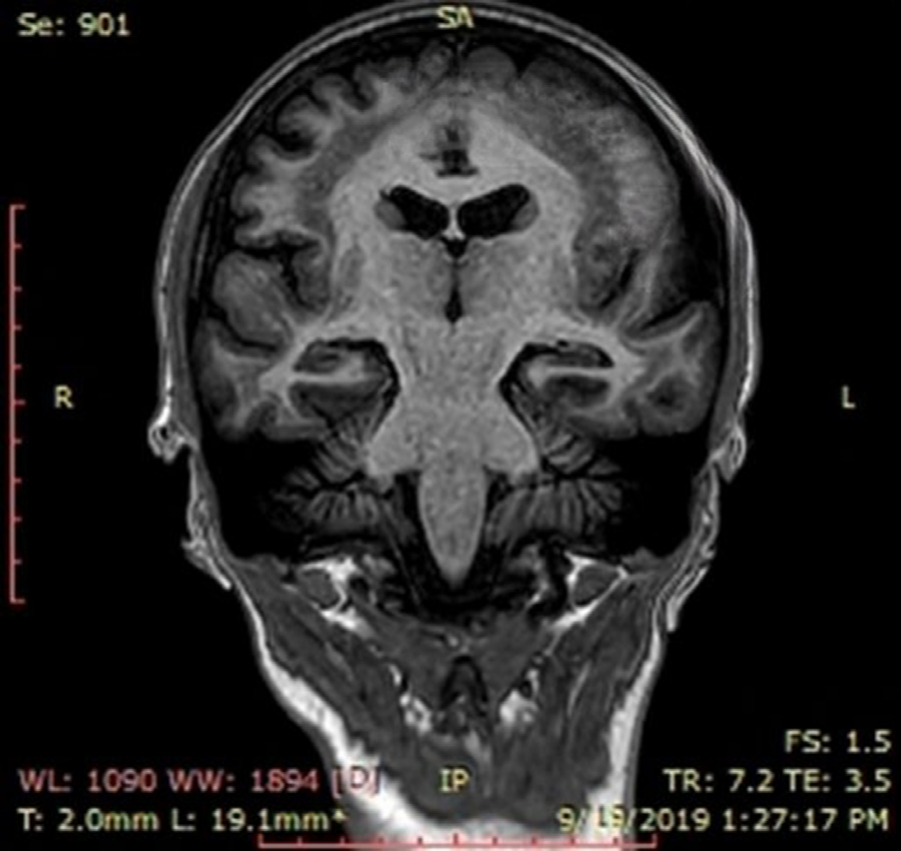
MRI brain T1WI 3D TFE Coronal images - The heterotopic grey matter almost paralleling the cortex is appreciated.

**Fig. 4:**
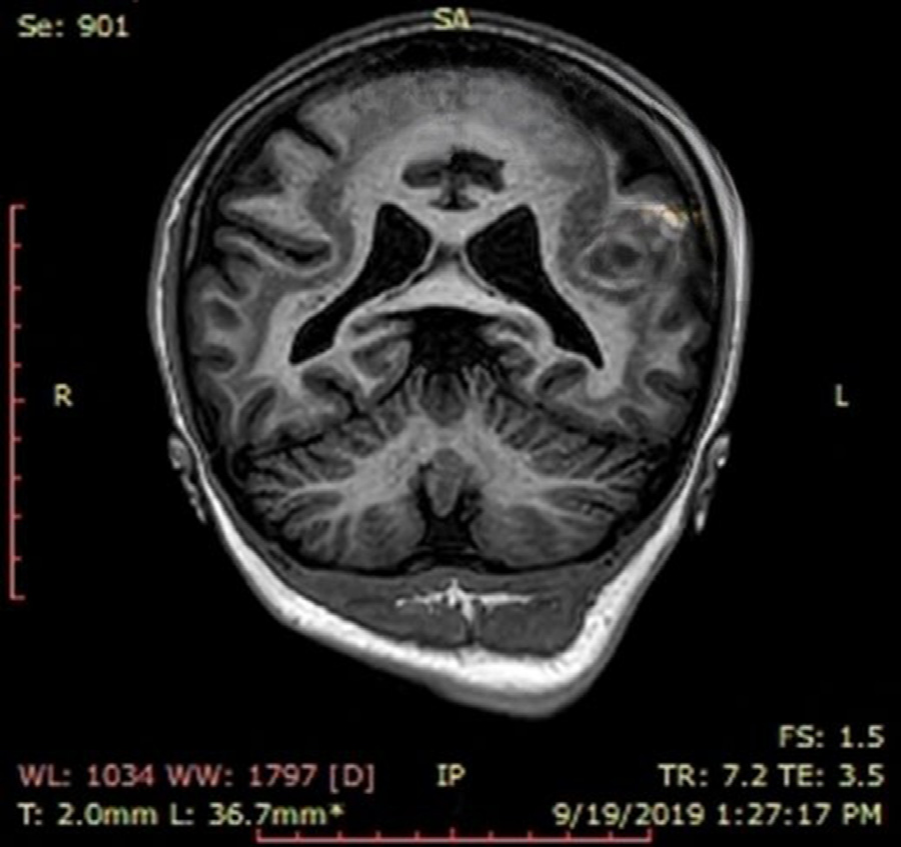
MRI brain T1WI 3D TFE Coronal images - The heterotopic grey matter almost paralleling the cortex is appreciated.

Figures 3 and 4 show MRI brain T1WI 3D TFE Coronal images—The heterotopic gray matter almost paralleling the cortex is better appreciated.

## Discussion

Neuronal migration abnormalities are chief cause of uncontrolled epilepsy and developmental delay in children and adolescents. Developmental disabilities in the recent few years have been reported to be increasing in children falling in age group between 3 and 17 years. Therefore, early diagnosis is crucial for sake of proper management and understanding of child disabilities and parent guidance. MRI is the imaging tool for accurate diagnosis so that not only symptomatic management of fits is done but the issue can be addressed in all aspects regarding parent counseling and providing proper speech and behavioral therapies aimed to improve patient compliance to cope up with his/her disabilities. Although such cases are rare, yet they have been reported and discussed in literature from different regions of the world. Ali Akbar Momen has reported first case of double cortex syndrome from Iran of a developmentally delayed female presenting with epilepsy [8]. More studies are also being conducted regarding double cortex syndrome in males too though it is uncommon in males. One such study was done by Mitsuhiro Kato on 2 males diagnosed with subcortical band heterotopia (SBH). There was heterozygous mutation for Asp50Lys or Arg39Stop in both the patients. On MRI, the characteristic imaging finding was present as seen in females [9]. Another study conducted by Ono et al. presented a mentally retarded Japanese boy with normal male karyotype, 46 XY diagnosed as double cortex syndrome [10]. An interesting study identified a somatic mosaicism for deletion of exon 4 in the DCX gene in a male patient resulting in prenatal diagnosis of subcortical band heterotopia([11]. Association of SBH with lissencephaly, pachygyria, and agyria has been reported in medical literature [12]. There is also associated reduction in patient functional capabilities, learning process, concentration, and social interaction [13,14]. Patients with lissencephaly have poor prognosis with short life span due to multiple environmental and developmental factors [15]. Our patient was achieving milestones normally when suddenly there was a decline noted in her speech and with the passage of time learning disabilities were observed. She had been suffering from seizures off and on for 8 years but was not diagnosed as her CT scan done at age of 7 years was unremarkable. It was on MRI that an accurate diagnosis was made.

## Conclusion

Double cortex syndrome is an uncommon and serious syndrome which needs to be addressed properly for sake of both patient and parents. Using advanced imaging technology, MRI, we can accurately diagnose the problem and ultimately direct the neurologist/physician toward a better plan of management for the patient, parent counseling regarding prognosis, future family planning, genetic counseling, and parent education about the significance of therapies needed for the improvement of patient. This approach will definitely have a positive impact on the patient life so that he/she can perform to their best possible role as a member of society.

## Ethical approval

Not required as we have acquired consent from the patient.

## Author contribution

All authors contributed equally.

## Patient consent

Written informed consent was obtained from the mother of patient for publication of this case report and accompanying images. A copy of the written consent is available for review by the Editor-in-Chief of this journal on request.

## Research registration

1. Name of the registry: NA.
2. Unique Identifying number or registration ID: NA.
3. Hyperlink to your specific registration (must be publicly accessible and will be checked): NA.

## Provenance and peer review

Not commissioned, externally peer reviewed.

## References

[1] Kaur S, Ghuman MS, Devarajan LJ (2015). A pediatric epilepsy classic: “Double cortex” syndrome. J Pediatr Neurosci.

[2] Haverfield EV, Whited AJ, Petras KS, Dobyns WB, Das S. (2009). Intragenic deletions and duplications of the LIS1 and DCX genes: a major disease-causing mechanism in lissencephaly and subcortical band heterotopia. Eur J Hum Genet.

[3] Granata T, Battaglia G, D'Incerti L, Franceschetti S, Zucca C, Savoiardo M (1994). Double cortex syndrome: electroclinical study of three cases. Ital J Neurol Sci.

[4] Dericioglu N, Oguz KK, Ergun EL, Tezer FI, Saygi S. (2008). Ictal/interictal EEG patterns and functional neuroimaging findings in subcortical band heterotopia: report of three cases and review of the literature. Clin EEG Neurosci.

[5] D'Agostino MD, Bernasconi A, Das S, Bastos A, Valerio RM, Palmini A (2002). Subcortical band heterotopia (SBH) in males: clinical, imaging and genetic findings in comparison with females. Brain.

[6] Parisi P, Miano S, Mei D, Paolino MC, Castaldo R, Villa MP. (2010). Diffuse subcortical band heterotopia, periodic limb movements during sleep and a novel “de novo” mutation in the DCX gene. Brain Dev.

[7] Gressens P, Passemard S, Sebag G, Chalard F, Laquerriere A., Squire LR (2009). Encyclopedia of neuroscience [Internet].

[8] Momen AA, Momen M. (2015). Double cortex syndrome (subcortical band heterotopia): a case report. Iran J Child Neurol.

[9] Kato M, Kanai M, Soma O, Takusa Y, Kimura T, Numakura C (2001). Mutation of the doublecortin gene in male patients with double cortex syndrome: somatic mosaicism detected by hair root analysis. Ann Neurol.

[10] Ono J, Mano T, Andermann E, Harada K, Sakurai K, Ikeda T (1997). Band heterotopia or double cortex in a male: bridging structures suggest abnormality of the radial glial guide system. Neurology.

[11] Quélin C, Saillour Y, Souville I, Poirier K, N'guyen-Morel MA, Vercueil L (2012). Mosaic DCX deletion causes subcortical band heterotopia in males. Neurogenetics.

[12] Leventer RJ, Pilz DT, Matsumoto N, Ledbetter DH, Dobyns WB. (2000). Lissencephaly and subcortical band heterotopia: molecular basis and diagnosis. Mol Med Today.

[13] Jacobs R, Anderson V, Harvey AS. (2001). Neuropsychological profile of a 9-year-old child with subcortical band heterotopia or “double cortex”. Dev Med Child Neurol.

[14] Bahi-Buisson N, Souville I, Fourniol FJ, Toussaint A, Moores CA, Houdusse A (2013). New insights into genotype-phenotype correlations for the doublecortin-related lissencephaly spectrum. Brain.

[15] Friocourt G, Marcorelles P, Saugier-Veber P, Quille ML, Marret S, Laquerrière A. (2011). Role of cytoskeletal abnormalities in the neuropathology and pathophysiology of type I lissencephaly. Acta Neuropathol.

